# The effect of mHealth application on improving childhood immunization coverage and timeliness: a case of the Gurage Zone, SNNPR, Ethiopia, a randomized controlled trial

**DOI:** 10.1186/s12889-025-25680-y

**Published:** 2025-11-22

**Authors:** Fisha Alebel Gebreeyesus, Bogale Chekole Temere, Baye Tsegaye Amlak, Tadesse Tsehay Tarekegn, Bisrat Zeleke Shiferaw, Mamo Solomon Emire, Omega Tolessa Geleta, Tamene Fetene Terefe, Agerie Ayinalem Mewahegn, Melkamu Senbeta Jimma, Ermias Sisay Chanie, Natnael Moges Misganaw, Katrina Lane-Krebs

**Affiliations:** 1 Department of Nursing, College of Medicine and Health Sciences, Wolkite University, PO Box 07, Wolkite, Ethiopia; 2 Department of Nursing, School of Nursing and Midwifery, College of Medicine and Health Sciences, Debre Birhan University, Debre Birhan, Ethiopia; 3 Department of Nursing, College of Medicine and Health Sciences, DebreMarkos University, DebreMarkos, Ethiopia; 4 Department of Nursing, College of Medicine and Health Sciences, Injibara University, Injibara, Ethiopia; 5 Department of Nursing, College of Health Sciences, Assosa University, PoBox 18, Assosa, Ethiopia; 6 Department of Pediatric and Neonatal Nursing, College of Health Sciences, Debre Tabor University, PO Box 272, Debre Tabor, Ethiopia; 7 School of Nursing, Midwifery and Social Sciences, Central Queensland University, Brisbane, Australia

**Keywords:** mHealth, Text message reminder, SMS reminder, Immunization, Vaccination, Gurage

## Abstract

**Introduction:**

Ethiopia has achieved remarkable decreases in under-5, infant, and neonatal mortality and has reached the target of MDG four three years ahead of the deadline, but child and infant health issues continue to result in high rates of mortality and morbidity. Therefore, to improve child and infant health services and accelerate the achievement of the Sustainable Development Goals, it is imperative that an innovative and cost-effective strategy be developed.

Previous studies have found that the implementation of digital health technology, particularly through mobile health technology, such as short message reminder systems, has enhanced vaccination coverage and increased the demand for routine vaccinations in children among low- and middle-income countries. However, there is a paucity of research on the effect of mHealth applications on improving routine childhood immunization coverage and timeliness in the Gurage Zone, SNNPR, Southwest Ethiopia.

**Methods:**

A randomized controlled trial with two arms and a parallel design was conducted with 408 mother-infant pairs. Using simple randomization, 204 mother-infant pairs from selected health institutions were assigned to the intervention group, which received routine health education and short message service reminders, and 204 mother-infant pairs to the control group, which received only routine health education. Mother-infant pairs were used as the randomization units and were randomized 1:1 into one of the two study arms. The data were exported to STATA version 14 statistical software for analysis after being cleaned and coded in EpiData version 4.1. The descriptive statistics were calculated first. To compare outcomes between study groups, log-binomial regression analyses and two-sample tests of proportions were employed. Variables were considered statistically significant when their *p*-value was less than 0.05.

**Results:**

Four hundred mother-infant pairs were enrolled in the trial, 200 of whom were in the control group and 200 of whom were in the intervention group. Eight mother-infant pairs were excluded from the analysis as they had been relocated outside the study area. Compared with the control group, a higher percentage of infants in the intervention group received Penta 1, 199 (99.5%) vs. 191 (95.5%), Penta 2, 192 (96%) vs. 157 (78.5%), and Penta 3, 172 (86%) vs. 133 (66.5%). At 14 weeks, the Penta 3 vaccine was administered to 172 (86%) and 133 (66.5%) infants in the intervention and control groups, respectively. The corresponding risk ratio (RR) was 1.29, with 95% CIs (1.15–1.45; *p* < 0.001). Additionally, the Penta 3 vaccine was administered on time to 159 (79.5%) and 123 (61.5%) infants in the intervention and usual care groups, respectively, with RRs of 1.29 and 95% CIs (1.13, 1.47; *p* < 0.001). In this study, 13.6% of infants in the intervention group and 31.4% in the control group dropped out of the Penta 3 immunization program. Despite a dropout rate exceeding an acceptable threshold, the intervention group demonstrated a significantly lower Penta 3 dropout rate than the control group.

**Conclusion:**

Our research showed that using a mobile health application through SMS reminder system can increase vaccination timeliness and coverage and strengthen the quality and effectiveness of an immunization program. The intervention substantially improved both the uptake and timely administration of the Penta vaccines compared to usual care. It is therefore recommended that the intervention approach be integrated into routine immunization programs to enhance vaccination coverage and timeliness among infants.

**Trial registration:**

Retrospective registration on the Pan African Clinical Trial Registry on March 24, 2025, PACTR202503488120509.

## Background

Child mortality reduction has been a core objective of the global health agenda, As set out in the Millennium Development Goals (MDGs) and the Sustainable Development Goals (SDGs) [1].

Immunization is essential for primary healthcare, an undeniable human right, and one of the best investments in health, saving between 3.5 million and 5 million lives each year from diseases such as measles, tetanus, diphtheria, and pertussis [2].

The impact of immunization encompasses more than just SDG 3, and it directly aids in achieving 14 of the 17 SDGs [3]. In 2017, a significant number of lives, approximately 1.5 million children, died due to vaccine-preventable diseases despite the proven advantages of immunization [4].

Worldwide, diseases that can be prevented by vaccines are the primary reason for illnesses and deaths in children under the age of five [5]. About 30% of child deaths under the age of five are attributed to diseases that could have been prevented by vaccines [6].

Children in southern Asia and sub-Saharan Africa face the highest risk of death and bear the greatest burden of child mortality [7]. Most child deaths occurred in low and middle-income countries, accounting for nearly 99% of total fatalities [8]. Vaccine-preventable illnesses are responsible for the majority of childhood mortality, making immunization coverage and timely administration particularly important in Sub-Saharan African countries [9].

As advised by the World Health Organization (WHO), children must receive all recommended vaccinations and be vaccinated on time to be fully protected against certain infections [10].

In 2018, nearly a third of African countries fell short of achieving 80% coverage for the third dose of the DTP vaccine [11, 12], while by that year, 129 countries had already surpassed 90% coverage for this vaccine dose [13]. Just 61% of Ethiopian children receive the same dose [14].

Despite being a measure of vaccination program quality, vaccination timeliness has not received as much attention as other program performance measures have [15]. For example, research conducted in Gambia revealed that just a third of infants received all vaccinations on time [16], whereas in Uganda, only 18% [17] 45.6% of children received all vaccinations on time [18]. Similarly, according to the 2019 Ethiopian mini demographic health survey, data showed that only 22% of the children had been immunized by the appropriate age, and just 4 out of every 10 infants had been fully vaccinated [14].

It is evident that low-income nations still have substantially lower immunization coverage and age-appropriateness compared to their counterparts in middle- and high-income nations [19].

Achieving the full impact of vaccines requires a combination of both high vaccination rates and timely administration [20]. Outbreaks of VPDs are on the rise as a result of suboptimal coverage and untimely childhood vaccination [21, 22]. As scheduled, timely vaccination coverage is necessary to effectively suppress and eradicate VPDs [23]. Despite notable advancements in Africa, overall coverage rates are still below the Global Vaccine Action Plan target [24].

Numerous factors are associated with children who are not vaccinated or have not received the recommended vaccinations: including communication gaps between childcare providers and healthcare workers, residing in rural areas, mothers’ forgetfulness, a lack of awareness regarding the need for follow-up doses, inadequate healthcare infrastructure, insufficient reminder services, limited access to healthcare services, transportation issues, and lengthy distances to healthcare facilities. Additionally, low health literacy among mothers and caregivers, coupled with a scarcity of trained staff members and a high staff turnover rate, has been implicated in these challenges [25–32].

A variety of policies and health system approaches have been introduced globally to promote immunization rates and on-time vaccination in various global regions. The strategies involve promoting mobile health technology by sending immunization reminders to mobile phones via SMS, and this approach has shown encouraging outcomes in increasing immunization rates [33–36].

Short message services, or SMSs, are text messaging components of mobile phones, web, and other mobile systems utilizing established protocols that enable users to exchange text messages [37]. SMS messages have been used to monitor and promote preventive health behaviors, improve attendance at treatment programs, remind clients of medical appointments, and provide a versatile solution that addresses a range of obstacles, including travel and intricate cross-cultural interactions [38, 39]. Text messaging via short message services is still one of the most widely used mobile services remains extremely popular, and it has become the world’s leading data transmission method, according to global statistics [40]. The Federal Ministry of Health has recognized and identified mobile health as a key driver of transformation in the healthcare sector, and has suggested that it could be a potential way to assist women throughout the vaccination period to increase the uptake of crucial maternity services [41, 42]. Vaccination coverage in Ethiopia has shown considerable regional disparities, with practically all regions falling short of the 80% target set by both the Global Vaccine Action Plan and the country’s own national goals [24, 43]. The Southern Nations, Nationalities, and Peoples’ Region (SNNPR) specifically recorded a low rate of complete immunization coverage of 38% in 2019 [44]. To date, no comprehensive immunization coverage assessment has taken place, with some districts experiencing recurring outbreaks of vaccine-preventable diseases, notably measles. The need for an innovative and cost-effective strategy is self-evident, one that, when properly implemented, will lead to enhanced adherence to childhood immunization schedules.

Studies have shown that mobile phone text messages in local languages, combined with automated phone calls, contribute to positive health behaviors, raise vaccination rates, and boost demand for routine childhood vaccination [33, 36]. Although mhealth technology is rapidly evolving, there is limited knowledge about its potential impact on improving immunization programs in Ethiopia. The main goal of this trial was to evaluate the effect of a mobile health (mHealth) application on improving childhood immunization coverage and timeliness in the Gurage Zone, SNNPR, Ethiopia, by conducting a randomized controlled trial. We hypothesized that a mother-infant pair receiving a mobile phone text messaging intervention would have a higher percentage of immunization coverage and timeliness, and a lower immunization dropout rate compared to a control group.

## Methods and materials

### Study area and period

The research was conducted in the public health facilities of the Gurage Zone located within the Southern Nations, Nationalities, and Peoples’ Region state. The capital of the Gurage Zone is Wolikite town, which is situated 260 km from Hawasa and 158 km southwest of Addis Ababa. A total of 5,932 square kilometers make up the Gurage Zone. With two municipalities and 20 woredas, the zone consists of a total of 443 kebeles, with 411 being rural and 32 being urban. In accordance with the CSA’s 2012 population prediction, the overall population totals 1,767,518, with nearly 15.6% (275,910) comprised of children below the age of five.

There are seven hospitals (one nongovernmental and six public hospitals), five primary hospitals, one general hospital, and one a single specialized comprehensive hospital. The zone is served by 79 health centers, including seven that are operated by NGOs, and 444 functioning health posts. The participants in the study were followed from 15 June 2021 until 21 December 2021.

### Study design

A two-armed, parallel, randomized controlled trial was employed. The research was conducted in accordance with the updated CONSORT guidelines for reporting parallel randomized controlled trials. The study was conducted in compliance with the CONSORT revised guidelines for the documentation of parallel randomized trials [45].

### Populations

#### Source population

Every mother-infant pair living within the Gurage zone that fulfills the specified selection criteria.

#### Study population

Mother-infant pairs who fulfilled the specified selection criteria within the chosen Gurage zonal health facilities. A target participation of over 400 mother-infant pairs was sought.

### Enrollment criteria

#### Inclusion criteria

- Enrollment was open to parents or caregivers whose child was less than 4 weeks old and had not yet received the first dose of the pentavalent vaccine.
- At least one member of the household or a caregiver should have a functioning mobile phone with an active connection, and
- Residents who are permanently living in the study area.

#### Exclusion criteria

- Mothers/caregiver who plans to attend a different center for the child’s remaining vaccination visits

### Sample size determination and sampling procedures

The main goal is to evaluate how well the interventions work to enhance vaccination rates and timeliness with a minimum absolute difference of 15 percentage points at each vaccination session relative to the control group.

The sample size was calculated based on a 50% immunization coverage rate due to the absence of prior studies, and an expected 15% increase in the intervention group (65%), with a desired 80% power, 5% type 1 error rate, and 20% anticipated loss to follow-up.

$$n=\frac{({Z}_{1-\alpha /2}+{Z}_{1-\beta }{)}^{2}\cdot [{p}_{1}(1-{p}_{1})+{p}_{2}(1-{p}_{2})]}{({p}_{1}-{p}_{2}{)}^{2}}$$

Where:

- $n$ = sample size per group
- ${p}_{1}$ = expected proportion in control group (50%)
- ${p}_{2}$ = expected proportion in intervention group (65%)
- ${Z}_{1-\alpha /2}$ = Z-score for the desired significance level (1.96 for 5% two-sided)
- ${Z}_{1-\beta }$ = Z-score for the desired power (0.84 for 80% power)
- $$\alpha$$ = significance level (0.05)
- $$\beta$$ = 1—power (80%)
- Minimum detectable effect size: 15%
- Lost to follow-up- 20%.

Previous studies have established that an effect size of 10% to 20% is considered clinically significant and relevant for shaping public health policy [46, 47]. A 15% difference between the intervention and control groups was selected, as it signifies a considerable public health effect and could motivate policymakers to adopt and incorporate the system within Ethiopia. The total number of samples in the final analysis was 408. As a result, the sample size in each group consisted of 204 mother-infant pairs.

### Enrollment and randomization

#### Recruitment process

Mother‒infant pairs who presented for an infant’s BCG immunization at the immunization units of the selected health institutions were first evaluated for eligibility by the research assistant and given a detailed description of the purpose of the study to the participant, and the research assistant was responsible for sequence generation and participant enrollment.

Following confirmation of eligibility, written consent was secured from participants, after which the mother-infant pairs were incorporated into the study, and ongoing follow-up arrangements were made with their respective health facilities. The infant’s medical history was assessed for enrollment if the parent or caregiver was interested in the research. Enrollment will cease once a total of 408 mother-infant pairs has been reached.

#### Randomization and allocation

Caregivers and mothers who attended vaccination at selected Gurage zonal health institutions were randomly assigned to either an intervention group, which included 204 mother-infant pairs that received reminders via short message service and routine health education, or a comparison group consisting of 204 mother-infant pairs that only received routine health education. The units of randomization were mother-infant pairs that were randomly assigned to one of two trial groups in a 1:1 ratio.

The randomization process was stratified by health institution to ensure that each institution had a proportional number of participants in both the intervention and control groups using sealed and opaque envelopes within each health facility separately, based on the client flow within each institution. We used sealed, opaque, identical, and small-sized envelopes for the random sequence generation to prevent knowledge of intervention assignment before the study participant’s enrolment. A research assistant ensured a random sequence generation by marking the study arms on paper, folding them, and placing them in an envelope. The sealed envelopes for both the intervention and control groups were then combined and shuffled for each health facility, ensuring the allocation sequence remained concealed.

### Intervention

A preliminary baseline survey of the mother-infant pairs was carried out, encompassing basic socio-demographic characteristics; factors related to maternal healthcare use, factors related to access, the respondent’s mobile phone details, and the preferred language for receiving text reminders, which were either English or Amharic.

The participants in the research were allocated randomly to either an intervention group or a control group at the start of the study via a simple randomization technique. Those who received routine health education without a short message service reminder and those who received both health education and a short message service reminder. The contents of health education delivered to mothers enrolled in the intervention group and control group were similar.

Mother‒infant pairs that participated in the study were followed for 24 weeks. The mothers and caregivers in the control group were notified of their upcoming appointment and received routine health education. The research assistant let the study participants know when they may expect to receive SMS text message alerts about routine vaccinations that included information, reminders, and proactive messages for those mothers grouped under the intervention group. The message was delivered one day, three days, or one week before the vaccine due date, as per the recipients’ preferences indicated in the baseline survey. The messages were sent at six, ten, and fourteen weeks, which corresponded to the three pentavalent vaccination doses. Reminders were sent via text message in English or Amharic, as per the mother’s preference.

The following was the translated message: “Immunization shields your child from deadly illnesses such as tuberculosis, polio, whooping cough, diphtheria, hepatitis, measles, pneumonia, and diarrhea.” The vaccinations for your child [name] are due at the closest [clinic] tomorrow, three days from now, or one week from now.

### Blinding

Due to the nature of the intervention (short message service and health education), it was impossible to blind the study participants as well as the data collectors. However, to prevent ascertainment bias during outcome ascertainment, health workers who recorded the vaccination status of infants and the statistician involved in data entry and analysis were blinded to study group allocations.

### Outcome ascertainment

Data for primary and secondary outcomes were determined from vaccination cards, retrospectively collected data, and the clinic’s immunization register during and at the end of the follow-up.

### Variables

#### Primary outcomes

Vaccination coverage for each vaccine.

On-time immunization coverage for each vaccine.

#### Secondary outcomes

Vaccine dropout rate among pentavalent vaccine recipients.

#### Independent variables

The sociodemographic features of caregivers and the child, maternal healthcare utilization factors, access-related factors, and mobile phone characteristics of the respondent.

### Operational definition

#### On-time vaccination for specific vaccines

Receipt of vaccination for specific vaccines should be administered within 4 days before and within 4 weeks after the recommended date as per the EPI schedule [32].

### Data collection instrument (tools) and procedure

The data were gathered using a pre-tested, structured questionnaire, which was administered through face-to-face interviews, and was originally developed following an extensive review of existing literature.

For the baseline and subsequent follow-up data collection, a total of fifteen [15] BSc nurses proficient in Amharic, the predominant local language in the study area, were utilized, and the process was overseen by three (MSc) nurses. The completed questionnaires were thoroughly reviewed by the principal investigator each day to ensure they were complete and consistent.

#### Data quality assurance and control

A preliminary baseline survey was carried out to guarantee the quality of the data which covered information on the child’s and caregivers’ basic sociodemographic characteristics, maternal health care utilization-related factors, access-related factors, the respondent’s mobile phone characteristics, mothers and caregivers’ readiness to answer questions, their child’s birth date, the child’s full names, the appropriateness of the questions, the preferred language for receiving messages (English or Amharic), and the preferred time for receiving SMS text.

For the sake of suitability and convenience in communicating with the study participants, the questionnaire, information sheet, and informed consent were translated from English into Amharic. These were then retranslated into English for analysis by experts in the two languages.

Training was provided to data collectors and supervisors on the data collection instruments and processes before data collection began, ensuring a shared understanding of the methods and procedures used. Additionally, throughout the entire follow-up, ongoing supervision was conducted.

#### Data processing and analysis

Data coded, entered, and cleaned in EpiData Version 4.1 and exported to STATA Version 14 for analysis. Initially, calculations of descriptive statistics were performed.

Proportions of vaccination coverage and timeliness for each vaccine were analyzed using descriptive statistics. Characteristics such as sociodemographics, mobile phone use, and immunization status were presented in terms of percentages for both the intervention and control groups.

Chi-square tests were used to evaluate the comparability of the data on sociodemographic factors and mobile phone use between the two groups, as well as vaccination status at 6, 10, and 14 weeks. The cut-off point for statistical significance is a *p*-value below 0.05. Proportions were used to display vaccination coverage and timeliness for each vaccine.

Absolute differences in the proportions of individuals with corresponding 95% confidence intervals were calculated to evaluate the changes in vaccination status between the two study groups. A two-sample test of proportions was utilized to evaluate the statistical significance of the absolute differences. The risk ratios for primary outcomes were evaluated and contrasted between the intervention and standard care groups using log-binomial regression analysis. In the subgroup analysis, a log-binomial model was employed, and interaction terms were examined to investigate possible effect modification. The effect was described using the relative risk with a corresponding confidence interval of 95%. The threshold for statistical significance in this study was set at 0.05.

#### Ethical clearance

Ethical clearance was secured from the Ethical Review Committee of the College of Medicine and Health Sciences at Wolkite University, reference number RCSUILC/08/2013.

Data collection took place after written permission was secured from the selected public health institutions. Mothers or caregivers were required to provide their informed written consent following detailed explanations of the study’s objective, purpose, confidentiality, benefits, and potential risks. The respondents’ right to opt out of the study at any time was respected, and complete confidentiality was guaranteed. The research was carried out in accordance with the Declaration of Helsinki.

## Result

### Enrollment of the study participants

**Fig. 1:**
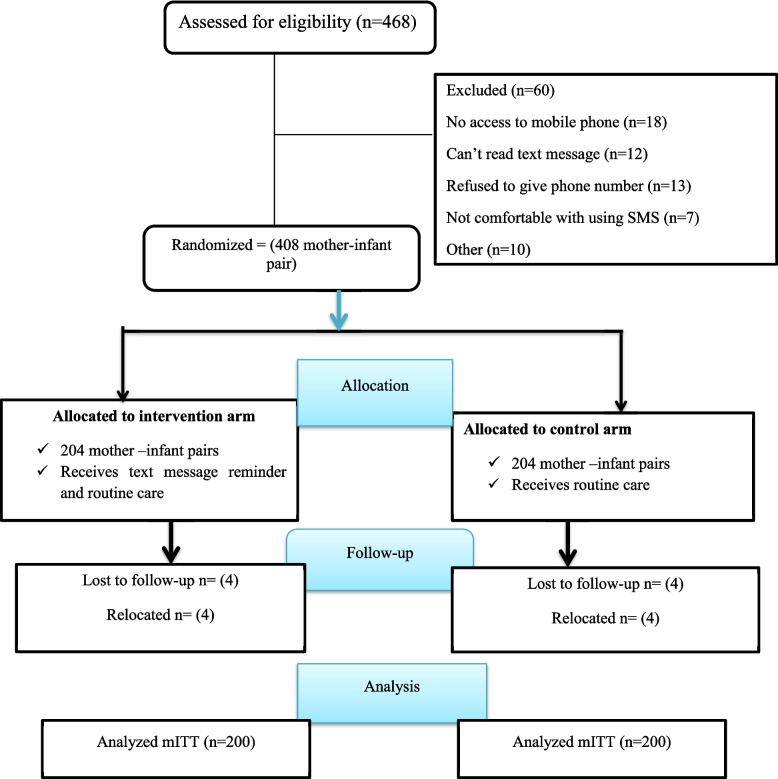
The CONSORT flow diagram of study participant enrollment, randomization, allocation, and analysis for the trial in Gurage zonal health institutions, Southwest, Ethiopia. 2022 (*N* = 400)

The baseline survey was conducted from June 15 to August 30, 2021, at which time 408 mothers and infants were enrolled in the study, and we followed them for 24 weeks. Out of the total number of mother-infant pairs enrolled in the study, 204 were randomly assigned to the intervention group, while the remaining 204 pairs were assigned to the control group. Analyses were conducted on 400 mother-infant pairs who were followed for a period of 6 months in both groups using the modified intention-to-treat analysis principle (Fig. 1). Eight mother-infant pairs were not included in the analysis because they were transferred out of the study area (Fig. 1).

### Sociodemographic characteristics

Initially, the study participants in both the intervention and control groups exhibited comparable characteristics. The chi-square test for baseline characteristics revealed no statistically significant difference between the usual care and intervention groups at the trial’s commencement. However, a difference in fathers’ occupational status and mothers’ educational status was observed.

Three-fifths of the mothers in the intervention group (60.5%) and control group (59.5%) were between 25 and 34 years of age. The mean ages of participants in the intervention and control groups were 26.16 ± 4.49 and 26.74 ± 4.76 years, respectively. Approximately half of the respondents (51.5%) in the intervention group and (53.5%) in the control group were Muslim religious followers. Housewives were the most prominent occupation in the intervention (63.0) and control group (61.0). Approximately one-third (33.5%) and 28.5% of mothers attained primary school education in the intervention and control group, respectively.

In the interventional and control groups, nearly all (97.5%) of the fathers were actively involved in caring for their infants. The mean ages of the fathers in the intervention group and the control group were 32.74 ± 5.79 and 33.77 ± 5.69 years, respectively. More than two-fifths (44.5%) of fathers in the intervention group and (36%) in the control group had attended college/university (Table 1).

**Table 1:** Baseline characteristics of the study participants enrolled in the trial in Gurage zonal health institutions, Southwest Ethiopia 2022 (*N* = 400)

Characteristic’s	Categories	Intervention group (*n* = 200)	Control group (*n* = 200)	*p*-value
Age of the mother	< = 24	68 (34)	65 (32.5)	0.357
	25–34	121 (60.5)	118 (59)	
	> = 35	9 (4.5)	16 (8.0)	
Relations of the respondent to the child	Mother	198 (99)	193 (96.5)	0.513
	Siblings	2 (1)	6 (3.0)	
	Grand mother		1 (0.5)	
Religion	Orthodox	82 (41)	76 (38)	0.806
	Muslim	103 (51.5)	107 (53.5)	
	Protestant	11 (5.5)	13 (6.5)	
	Catholic	4 (2)	3 (1.5)	
	Other		1 (0.5)	
Marital status	Married	200 (100)	199 (99.5)	0.317
	Single		1 (0.5)	
Occupation of mothers	Housewife	126 (63)	122 (61.0)	0.701
	Merchant	21 (10.5)	22 (11.0)	
	Government employed	46 (23)	43 (21.5)	
	Daily worker	6 (3)	12 (6.0)	
	Student	1 (0.5)	1 (0.5)	
Ethnicity	Gurage	146 (73)	140 (70.0)	0.785
	Oromo	38 (19)	37 (18.5)	
	Amhara	7 (3.5)	8 (4.0)	
	Tigre	1 (0.5)	2 (1.0)	
	Others	8 (4)	13 (6.5)	
Educational status of mothers	Not read and write	23 (11.5)	34 (17.0)	
	Read and write	11 (5.5)	31 (15.5)	0.005
	Primary school	67 (33.5)	57 (28.5)	
	High school	46 (23)	37 (18.5)	
	College/university	53 (26.5)	41 (20.5)	
Residence	Urban	179 (89.5)	174 (87.0)	0.438
	Rular	21 (10.5)	26 (13.0)	
Age of the father	18–29	46 (23)	34 (17.0)	0.134
	>= 30	154 (77.0)	166 (83.0)	
Fathers educational status	Not read and write	16 (8.0)	30 (15.0)	0.184
	Read and write	11 (5.5)	12 (6.0)	
	Primary school	41 (20.5)	40 (20.0)	
	High school	43 (21.5)	46 (23.0)	
	College/university	89 (44.5)	72 (36.0)	
Fathers employment status	Farmer	18 (9.0)	22 (11.0)	0.016
	Merchant	38 (19.0)	59 (29.5)	
	Government employed	80 (40,0)	52 (26.0)	
	Daily worker	61 (30.5)	61 (30.5)	
	Student	3 (1.50)	3 (1.5)	
	Other		3 (1.5)	
Fathers involved in caring of the infant	Yes	195 (97.5)	195 (97.5)	1.00
	No	5 (2.5)	5 (2.5)	
Numbers of family live in the household	< 5	163 (81.5)	167 (83.5)	0.599
	≥ 5	37 (18.5)	33 (16.5)	
Number of live birth	1	82 (41.0)	78 (39.0)	0.683
	≥ 2	118 (59.0)	122 (61.0)	
Family living conditions	Both father and mother alive	198 (99.0)	199 (99.50	0.223
	Mother only	2 (1.0)	1 (0.5)	
	Father only			
Sex of the child	Male	101 (50.5)	102 (51.0)	0.920
	Female	99 (49.5)	98 (49.0)	
Birth order of the child	First	80 (40.0)	81 (40.5)	0.986
	Second or later	113 (56.5)	114 (57.0)	
Average monthly income (*n* = 354)	≤ 1000 ETB	11 (6.1)	16 (9.1)	0.74
	1001–3000 ETB	48 (26.8)	42 (24)	
	3001–5000 ETB	40 (22.3)	48 (27.4)	
	> 5000 ETB	80 (44.7)	69(39.4)	

### Maternal health care utilization-related factors

In this research, a majority of participants in the intervention group (56%) and those receiving standard care (52.5%) had four antenatal care visits during their pregnancy. Approximately four-fifths (81.5%) and 79.5% of the patients in the intervention and control groups, respectively, had PNC follow-up. More than one-third of the participants in both groups had at least one PNC visit. In addition, more than 80% of the participants in the intervention group and 78% of those in the control group received two or more TT vaccinations. The majority of the mothers (94%) in both the interventional and control groups had planned pregnancies (Table 2).

**Table 2:** Maternal health care utilization characteristics of the study participants enrolled in the trial in Gurage zonal health institutions, Southwest Ethiopia 2022 (*N* = 400)

Characteristics	Categories	Intervention group (*n* = 200) (%)	Control group (*n* = 200) (%)	*p*-value
ANC follow-up	Yes	199 (99.5)	196 (98.0)	0.177
	No	1 (0.5)	4 (2.0)	
Frequency of ANC visit	1	8 (4.0)	16 (8.0)	0.191
	2	39 (19.5)	47 (23.5)	
	3	41 (20.5)	32 (16.0)	
	4	112 (56.0)	105 (52.5)	
PNC follow-up	Yes	163 (81.5)	159 (79.5)	0.614
	No	37 (18.5)	41 (20.5)	
Frequency of PNC visit	1	75 (37.5)	71 (35.5)	0.787
	2	58 (29.0)	62 (31.0)	
	3	30 (15.0)	26 (13.0)	
TT vaccination	Yes	188 (94.0)	193 (96.5)	0.240
	No	12 (6.0)	7 (3.5)	
Frequency of TT vaccination	1	26 (13.0)	17 (8.5)	0.062
	2	116 (58.0)	109 (54.5)	
	≥ 3	47 (23.5)	67 (33.5)	
Was your pregnancy planned	Yes	189 (94.5)	188 (94.0)	0.830
	No	11 (5.5)	12 (6.0)	

### Access-related factors

In this research, over 90% of the participants in the intervention group and 174 (87%) from the control group travelled less than 30 min to access immunization services, and nearly equal proportions (71%) of mothers had short waiting times to receive immunization services. The most widely used mode of transportation in both groups is the taxi or Bajaj. Health education was provided at the health facility in nearly equal proportions in both the intervention and the control group. Home visits were undertaken by health workers, and nearly two-thirds (64%) of mothers in the intervention groups and more than three-fifths (61.5%) in the control group (Table 3).

**Table 3:** Access-related factors for respondent’s whose children receiving immunization service in Gurage zonal health institutions, Southwest Ethiopia 2022 (*N* = 400)

Characteristic’s	Categories	Intervention group (*n* = 200) (%)	Controlgroup (*n* = 200) (%)
Distance to reach to health facility	< 15 min	67 (33.5)	68 (34.0)
	15–30 min	116 (58.0)	106 (53.0)
	> 30 min	17 (8.5)	26 (13.0)
Waiting time	< 15 min	63 (31.5)	64 (32.0)
	15–30 min	81 (40.5)	77 (38.5)
	30–60 min	31 (15.5)	32 (16.0)
	> 60 min	25 (12.5)	27 (13.5)
How do you get to the health facility	Foot/walking	53 (26.5)	65 (32.5)
	Bajaj/taxi	144 (72.0)	133 (66.5)
	Other	3 (1.5)	2 (1.0)
Transport cost	1–10 ETB	97 (65.5)	88 (67.2)
	11–20 ETB	40 (27)	34 (24.4)
	> 20 ETB	10 (7.5)	11 (8.4)
Health education at the health facility	Yes	169 (84.5)	173 (86.5)
	No	31 (15.5)	27 (13.5)
Home visit by health workers	Yes	128 (64.0)	123 (61.5)
	No	72 (36.0)	77 (38.5)
Readiness of vaccines/supplies at the health facility	Yes	162 (81.0)	168 (84.0)
	No	38 (19.0)	32 (16.0)

### Mobile phone characteristics of the study participants

The majority of respondents in both the intervention group (84.5%) and the control group (80.55%) possessed their own working mobile phone. In the intervention group, 75.5% of mothers received SMS reminders. Mornings followed by afternoon time periods are the ideal time to receive SMS reminders. Approximately two-thirds (63.0%) of mothers preferred SMS reminders to be sent one day before the due date, followed by 29.5% and 7.5% three days and seven days before appointments, respectively. In addition, more than three-fifths (62.5%) of the study participants required just one SMS reminder, whereas 34% of the participants required at least two SMS reminders to remember the vaccination appointment (Table 4).

**Table 4:** Mobile phone characteristics of study participants in Gurage zone, Southwest Ethiopia, 2022 (*n* = 400)

Variables	Categories	Intervention group (*n* = 200)
Mobile phone access	Shared	31 (15.5)
	Owen phone	169 (84.5)
Receipt of SMS reminder	Mother	151 (75.5)
	Father	44 (22.0)
	Guardian	1 (0.5)
	Other	4 (2.0)
Number of working mobile phones present/owned in the home	1	61 (30.5)
	2	131 (65.5)
	≥ 3	8 (4.0)
For how long you have had your current mobile phone being used in the study?	< 6 month	30 (15.0)
	6–12 month	56 (28.0)
	1–2 year	61 (30.5)
	> 2 year	53 (26.5)
best time of the day to receive text message reminder	Night	16 (8.0)
	Morning	106 (53.0)
	Afternoon	63 (31.5)
	Evening	15 (7.5)
Preferred language for Immunization SMS reminder	Amharic	193 (96.5)
	English	4 (2.0)
	Guragegna	3 (1.5)
Preferred time of SMS reminder	A day before appointment	126 (63.0)
	Three days before appointment	59 (29.5)
	A week before appointment	15 (7.5)
How many SMS reminders do you need to remember vaccination appointments?	1	125 (62.5)
	2	68 (34.0)
	≥ 3	7 (3.5)

### Effect of mobile phone text message reminders on receipt and timely receipt of the Penta 3 vaccine

The percentage of infants who received the third dose of vaccination in the intervention group was substantially higher than that in the group receiving usual care (86% vs. 66.5%, *P* < 0.001).

The timely administration of the Penta3 vaccine among the study participants revealed a significant difference between the intervention group and the control group. Specifically, the intervention group had a significantly higher rate of timely receipt of the third dose of Penta3 vaccine at 79.5% compared to the control group at 61.5% (*P* < 0.001). Furthermore, the intervention group also showed a significantly higher rate of timely receipt of the Penta2 vaccine at 86.5% compared to the control group at 75% (*P* < 0.003) (Table 5).

**Table 5:** Effect of mobile phone text message reminders on receipt and timely receipt of Penta 3 vaccine among infants in Gurage Zone, Ethiopia in 2022 (*N* = 400)

Vaccination status	Intervention group (*n* = 200) *n* (%)	Usual care group (*n* = 200), *n* (%)	Absolute difference (%) (95% CI)	*P* value
Penta 3coverage	172(86)	133(66.5)	19.5(11.4,27.6)	< 0.001
Timely taking of Penta 3	159(79.5)	123(61.5)	18(9.2,26.8)	< 0.001

### Effect of mobile phone text message reminders on specific vaccinations

The pentavalent vaccination coverage was used as an indicator to assess the specific vaccination coverage for the doses administered during the schedules at six weeks, ten weeks, and fourteen weeks. The research found that nearly all, or 99.5%, of the babies in the intervention group, and 95.5% of those in the control group, received the Penta1 immunization (*p* = 0.017). During the tenth week, the Penta 2 coverage in the group receiving the intervention was substantially higher than in the group receiving usual care, with 96% compared to 78.5% (*P* < 0.001). At fourteen weeks, the proportion of infants receiving the Penta3 vaccine in the intervention group was substantially higher than that of infants in the control group (86% versus 66.5%, respectively; *p* < 0.001) (Table 6).

**Table 6:** Effect of mobile phone text message reminders on receipt of specific vaccines in Gurage Zone, Ethiopia in 2022 (*N* = 400)

Vaccination status	Intervention group (*n* = 200) *n* (%)	Usual care group (*n* = 200), *n* (%)	Absolute difference (%) (95% CI)	*P* value
Penta 1	199(99.5)	191(95.5)	3.5(0.06, 6.4)	0.017
Penta 2	192(96)	157(78.5)	17.5(11.2,23.8)	< 0.001
Penta 3	172(86)	133(66.5)	19.5(11.4,27.6)	< 0.001

According to our study results, 13.6% of infants in the intervention group and 31.4% in the control group dropped out of the Penta 3 immunization program. Despite a dropout rate exceeding an acceptable threshold, the intervention group demonstrated a significantly lower Penta 3 dropout rate than the control group.

At 6 weeks, SMS reminders were not statistically significant at the 5% significance level. 3.5% (95% CI: (0.06–6.4)).

At 10 weeks, the risk difference between the group receiving SMS reminders and the control group was 17.5% (95%: 11.2–23.8). The risk difference between the group that received SMS reminders and the control group at 14 weeks was 19.5% (95% CI: (11.4–27.6)).

Approximately 18% of the children immunized in the intervention group were attributed to SMS reminders and may not have been immunized if SMS reminders were not used at the 10-week interval. Similarly, about 20% of the children vaccinated in the SMS intervention group may be attributed to SMS reminders, who likely would not have been vaccinated if SMS reminders had not been used at 14-week intervals.

### Effect of mobile phone text message reminders on the timely receipt of specific vaccines

This study found that 99% of infants in the intervention group and 95% in the control group were given the pentavalent vaccination timely.

In the intervention group, a significantly higher proportion of infants (86.5%) received the Penta2 vaccination on time, in contrast to the control group, where 75% of infants received it on time. The result was statistically significant, with a *P*-value of 0.003. Moreover, a substantially higher percentage of infants participating in the intervention group received their Penta 3 vaccinations on time, in comparison to those in the control group (79.5% vs. 61.5%, respectively; *p*-value < 0.001) (Table 7).

**Table 7:** Effect of mobile phone text message reminders on timely receipt of specific vaccines in Gurage Zone, Ethiopia in 2022 (*N* = 400)

Vaccination status	Intervention group (*n* = 200) *n* (%)	Usual care group (*n* = 200), *n* (%)	Absolute difference (%) (95% CI)	*P* value
Penta 1	198(99)	190(95)	4(0.68, 7.3)	0.018
Penta 2	173(86.5)	150(75)	11.5(3.9,19.1)	0.003
Penta 3	159(79.5)	123(61.5)	18(9.2,26.8)	< 0.001

At six weeks, SMS reminders were not statistically significant at the 5% significance level, with 4% (95% CI: 0.68–7.3). At 10 weeks, the risk difference between participants who received SMS reminders and those in the control group was 11.5% (95% CI: 3.9–19.1). Similarly, at 14 weeks, the risk difference between participants who received SMS reminders and those in the control group was 18% (95% CI: 9.2–26.8).

About 12% of children vaccinated in the intervention group may be attributed to the use of SMS reminders, and, likely, these children would not have been immunized at 10 weeks without the SMS reminders. In the same way, 18% of the children vaccinated in the SMS intervention group may be attributed to the use of SMS reminders, which probably would not have been vaccinated if these reminders had not been implemented at 14 weeks. We authors acknowledged that the findings may not be generalized beyond the study setting without further implementation research.

### Log binomial regression analysis of the effect of the intervention on study outcomes (receipt and timely receipt of the Penta3 vaccine)

A log-binomial regression analysis was used to assess the effects of mobile phone text reminders on the receipt and timely receipt of the Penta3 vaccine. Caregivers in the intervention group had a 29% higher likelihood of vaccinating their infants than caregivers in the control group (RR 1.29, 95% CI: 1.15, 1.45).

The effect of mobile phone text message reminders on receiving the Penta3 vaccine on time was comparable to its effect on Penta3 vaccination rates.

Mothers in the intervention group were 29% more likely to vaccinate their infants with the third dose of vaccines on time compared to mothers enrolled in the control group. (RR 1.29, 95% CI: 1.13, 1.47) (Table 8).

**Table 8:** Log-binomial regression analysis of the effect of mobile phone text message reminders on the study outcomes in Gurage Zone, Ethiopia in 2022 (*N* = 400)

Vaccination status	Intervention group (*n* = 200) *n* (%)	Usual care group (*n* = 200), *n* (%)	Risk ratio (95% CI)	*P* value
Penta 3 coverage	172(86)	133(66.5)	1.29(1.15,1.45)	< 0.001
Timely receipt of Penta 3	159(79.5)	123(61.5)	1.29(1.13,1.47)	< 0.001

### Subgroup analysis of the effects of mobile phone text message reminders on timely receipt and coverage of the Penta-3 vaccine

An interaction test was performed to observe the effects on Penta-3 vaccine coverage across the subgroups of the sociodemographic variables. According to the subgroup analysis, intervention effects were significantly greater for mothers with a family size of less than 5 (RR: 1.32, 95% CI: 1.17, 1.49), mothers from rural residential areas (RR: 2.23, 95% CI: 1.33, 3.74), and mothers aged 25–35 (RR: 1.32, 95% CI: 1.13, 1.54) (Table 9).

**Table 9:** Subgroup analysis of the effect of mobile phone text message reminders on receipt of Penta-3 vaccine in Gurage Zone, Ethiopia, 2022

Baseline Characteristics	Intervention group (*n* = 172), *n* (%)	Usual care group (*n* = 133), *n* (%)	Stratum-specific risk ratio (95% CI)	*P*-value
Age of the mother				
< = 24	60(88.2)	47(72.3)	1.22(1.03,1.45)	0.025
25–35	103(85.1)	76(64.4)	1.32(1.13, 1.54)	< 0.001
>= 35	7(77.8)	9(56.3)	1.38(.79,2.41)	0.25
Residence				
Urban	154(86)	123(70.7)	1.22(1.09, 1.36)	0.001
Rural	18(85.7)	10(38.5)	2.23(1.33, 3.74)	0.002
Family income				
< = 5000	84(84.9)	59(55.7)	1.52(1.26, 1.84)	< 0.001
> 5000	69(86.3)	53(76.8)	1.22(0.96, 1.31)	0.146
Family size				
< 5	143(87.7)	111(66.5)	1.32(1.17,1.49)	< 0.001
>= 5	29(78.4)	22(66.7)	1.17(.88,1.58)	0.28
Distance from the health facility				
< 15 min	56(83.6)	58(85.3)	0.98(0.85–1.13)	0.784
15–30 min	101(87.1	64(60.4)	1.44(1.22–1.71)	< 0.001
> 30	15(88.2)	11(42.3)	2.09(1.29–3.37)	0.003
Educational status of the mother				
Not read and write	20(87)	18(52.9)	1.64(1.15, 2.34)	0.006
Read and write	10(90.9)	21(67.7)	1.34(0.99, 1.82)	0.06
Primary school(1–8)	56(83.6)	40(70.2)	1.19(.97, 1.45)	0.086
Highschool (9–12)	43(93.5)	26(70.3)	1.33(1.06,1.66)	0.012
College/university	43(81.1)	23(68.3)	1.19(0.93 1.52)	0.169
Own mobile phone				
Yes	157(88.2)	121(66.9)	1.32(1.17, 1.48)	< 0.001
No	15(68.2)	12(63.2)	1.08(0.68, 1.68)	0.737

Intervention effects on timely receipt of the Penta-3 vaccine were also significantly greater for mothers with a family size of less than 5 (RR: 1.33, 95% CI: 1.16, 1.53), mothers from rural residential areas (RR: 3.01, 95% CI: 1.54, 5.85), and mothers who owned mobile phones (RR: 1.31, 95% CI: 1.14, 1.5) (Table 10).

**Table 10:** Subgroup analysis of the effect of mobile phone text message reminders on timely receipt of Penta-3 vaccine in Gurage Zone, Ethiopia, 2022

Baseline Characteristics	Intervention group (*n* = 159), n (%)	Usual care group (*n* = 123), n (%)	Stratum-specific risk ratio (95% CI)	*P*-value
Age of the mother				
< = 24	59(86.8)	43(66.1)	1.31(1.08, 1.60)	0.007
25–35	92(76)	72(61)	1.25(1.05, 1.49)	0.014
>= 35	6(66.7)	7(43.7)	1.52(.74, 3.14)	0.253
Residence				
Urban	142(79.3)	116(66.7)	1.19(1.05,1.35)	0.008
Rural	17(80.9)	7(26.9)	3.01(1.54,5.85)	0.001
Family income				
< = 5000	77(77.8)	56(52.8)	1.47(1.19, 1.81)	< 0.001
> 5000	64(81)	50(72.5)	1.12(0.93, 1.34)	0.226
Family size				
< 5	134(82.2)	103(61.7)	1.33(1.16, 1.53)	< 0.001
>= 5	25(67.6)	20(60.6)	1.11(0.78,1.59)	0.547
Distance from the health facility				
< 15 min	48(71.6)	56(82.3)	0.87(0.721.05)	0.143
15–30 min	97(83.6)	59(55.7)	1.50(1.24, 1.81)	< 0.001
> 30	14(82.3)	8(30.8)	2.68(1.44, 4.96)	0.002
Educationalstatusofthe mother				
Not read and write	18(78.3)	17(50)	1.57(1.05,2.33)	0.028
Read and write	11(100)	18(58.1)		
Primary school (1–8)	50(74.6)	37(64.9)	1.15(0.91, 1.45)	0.248
High school (9–12)	39(84.8)	23(62.2)	1.36(1.03, 1.80)	0.03
College/university	41(77.4)	28(68.3)	1.13(0.88,1.46)	0.337
Own mobile phone				
Yes	144(80.9)	112(61.9)	1.31(1.14, 1.5)	< 0.001
No	15(68.2)	11(57.9)	1.18(0.73, 1.9)	0.5

## Discussion

Childhood immunization is a critical public health intervention to prevent vaccine-preventable diseases. Despite efforts to improve coverage, many regions, including the Gurage Zone in SNNPR, Ethiopia, still face challenges in achieving timely and complete vaccination. This randomized controlled trial aimed to evaluate the effect of mHealth application on improving childhood immunization coverage and timeliness in this setting.

In this study, a substantial number of caregivers reported that short message service reminders were successful in increasing immunization coverage and timeliness and in reducing the number of dropouts from vaccinations at the selected health institutions. The immunization rates were substantially higher among individuals who received SMS reminders compared to those who enrolled in the control group.

The vaccination coverage was higher by 20% in the intervention group compared to the control group. This increment is attributed to SMS reminders being sent at 14 weeks. Similarly, about 18% of the children in the intervention group received their vaccinations on time, thanks to SMS reminders, which suggests that without these reminders, they may not have been vaccinated at 14 weeks. Similar results were found in a study conducted in Gondar, Ethiopia, which showed that 9% of children who received vaccinations in the intervention group, as well as 19.5% of those vaccinated on time, may not have been immunized if SMS reminders had not been sent at 14-week intervals [48]. Research conducted in rural Kenya found that combining SMS reminders with incentives led to substantial improvements in the timeliness of vaccinations [49]. Similarly, a study in Zimbabwe found that roughly 16% of the children in the intervention group received vaccinations thanks to SMS, which would not have been possible if these reminders weren’t sent at 14-week intervals [37]. Women in South Africa who received SMS interventions were more likely to complete the continuum of maternal and infant health care [50], in rural Nigeria [51, 52], Côte d’Ivoire [59], Pakistan [35], Guatemala [53], and Bangladesh [54]. It has been shown that SMS text reminders can increase childhood vaccination rates.

In this study, although mobile reminders increased immunization coverage and timeliness, overall compliance was suboptimal. Between 14% and 34.5% of infants in the interventional and usual care groups did not receive Penta3 vaccination, and 20% in the interventional group and 38.5% in the standard care group did not receive Penta3 vaccination on time, which implies that a large percentage of infants were not immunized or protected against the consequences of VPDs.

Published research has demonstrated that boosting immunization rates accelerates vaccination timeliness [39], yet inconsistent data also indicate higher instances of untimely vaccinations in various settings. In studies carried out in Pakistan (58%) [35], Nigeria (69%) [52],Kenya (74%) [49], Vietnam (77.2%) [55], Zimbabwe (82%) [37], Nigeria (84%) [56], and Gondar, Ethiopia (88.7%) [48]. The discrepancy can be attributed to variations in study settings, demographic characteristics of the participants, and definitions of timeliness. The consequence of untimely vaccination is that children with incomplete or delayed vaccination schedules are exposed to an increased risk of vaccine-preventable diseases [57]. The timely administration of vaccines is widely regarded as a key metric for evaluating the effectiveness of local immunization initiatives worldwide [58].

### Strengths and limitations

A key aspect of our study was the randomized design with comparable groups at baseline, and it is the initial investigation to assess the impact of SMS reminders on vaccination rates and timeliness in southern Ethiopia. Our study also has certain limitations, as the authors were unable to track the delivery status of the SMS messages, as the system only sent in one direction.

The study excluded caregivers who did not have access to mobile phones, which may have resulted in a selection bias at the onset of the study, and due to constraints of time and resources, we were only able to follow up with our study participants until the third dose of Pentavalent vaccine, which makes it challenging to forecast whether we would observe a similar effect of our intervention for the Measles 1 and 2 vaccinations. The trial should have been registered prospectively, but it was instead registered retrospectively in March 2025 due to the author’s unfamiliarity with the importance of prospective registration.

## Conclusion

Our research showed that implementing an SMS reminder system can enhance immunization rates and ensure vaccinations are administered on time, ultimately boosting the quality and efficiency of an immunization programme. The intervention group had higher immunization coverage and timeliness compared to the usual care group. The observed increase can likely be attributed to the implementation of SMS reminders. Based on our research, it is highly advisable to expand this initiative to establish a comprehensive nationwide body of evidence across multiple regions and districts to enhance national vaccination rates and timeliness, and allow policymakers to incorporate mobile health strategies into the current national vaccination plan [59].

## Data Availability

The data and/or code will be made available on request by contacting the author.

## Abbreviations

BCG: Bacillus Calumet Guerin
DPT: Diphtheria, Pertussis, and Tetanus
EDHS: Ethiopian Demographic and Health Survey
EMDHS: Ethiopian Mini Demographic and Health Survey
EPI: Expanded Program on Immunization
GAVI: Global Alliance for Vaccine and Immunization
Hib: Haemophilus Influenza Type B
IPV: Inactivated Polio Vaccine
IRB: Institutional Review Board
NGO: Non-Governmental Organization
OPV: Oral Polio Vaccine
PCV: Pneumococcus Vaccine
SNNP: South Nation Nationality and People
UNICEF: United Nations Children Fund
VPDs: Vaccine Preventable Diseases
WHO: World Health Organization
